# Elderly patients with complex health problems in the care trajectory: a qualitative case study

**DOI:** 10.1186/s12913-020-05437-6

**Published:** 2020-06-29

**Authors:** Marianne Kumlin, Geir Vegar Berg, Kari Kvigne, Ragnhild Hellesø

**Affiliations:** 1 Inland Norway University of Applied Sciences, Elverum, Norway; 2 Innlandet Hospital Trust, Lillehammer, Norway; 3 Department of Nursing Science, Faculty of Medicine, Institute of Health and Society, University of Oslo, Oslo, Norway; 4 Department of Health Sciences, NTNU, Faculty of Medicine and Health Sciences, Gjøvik, Norway

**Keywords:** Care trajectory, Complex health problem, Elderly, Care pathway

## Abstract

**Background:**

Elderly patients with multiple health problems often experience disease complications and functional failure, resulting in a need for health care across different health care systems during care trajectory. The patients’ perspective of the care trajectory has been insufficiently described, and thus there is a need for new insights and understanding. The study aims to explore how elderly patients with complex health problems engage in and interact with their care trajectory across different health care systems where several health care personnel are involved.

**Methods:**

The study had an explorative design with a qualitative multi-case approach. Eleven patients (*n* = 11) aged 65–91 years participated. Patients were recruited from two hospitals in Norway. Observations and repeated interviews were conducted during patients’ hospital stays, discharge and after they returned to their homes. A thematic analysis method was undertaken.

**Results:**

Patients engaged and positioned themselves in the care trajectory according to three identified themes: 1) the patients constantly considered opportunities and alternatives for handling the different challenges and situations they faced; 2) patients searched for appropriate alliance partners to support them and 3) patients sometimes circumvented the health care initiation of planned steps and took different directions in their care trajectory.

**Conclusions:**

The patients’ considerations of their health care needs and adjustments to living arrangements are constant throughout care trajectories. These considerations are often long term, and the patient engagement in and management of their care trajectory is not associated with particular times or situations. Achieving consistency between the health care system and the patient’s pace in the decision-making process may lead to a more appropriate level of health care in line with the patient’s preferences and goals.

## Background

The World Health Organization [1] highlighted the need to implement an integrated people-centred health service, particularly for people with chronic or complex health conditions in need of care and support. Elderly persons with multiple health problems often experience disease complications and functional failure, resulting in a need for health care across different levels of care and social services. It has been shown that such care trajectories can be complex when many health and social personnel are involved [2, 3].

Several terms have been used to describe patients’ needs that span levels of health care system, including care pathways, clinical pathways, critical pathways, care trajectories, standardised patient pathways and care bundles. The term care pathway can be defined as the management of care and chronological activities of a health care process for a well-defined group of patients during a well-defined period of time [4]. Standardised care pathways have been suggested as a solution for ensuring patient safety, improving risk-adjusted patient outcomes, increasing patient satisfaction and optimizing the use of resources [5]. Nevertheless, studies have shown that standardised care pathways are more effective in contexts with predictable care trajectories and low uncertainty and complexity [4, 6, 7]. At present, health care authorities have an increased demand for patient treatment standardisation and patient treatment individualisation. Standardised care pathways promote procedures and standardised activities. However, questions have been asked if these pathways are a risk to patient preferences and if individual needs will receive less attention [8, 9].

In this study, we chose the term care trajectory that is commonly used to describe a patient’s journey through the health care system. According to Allen et al. [10], the term refers to ‘the unfolding of patients health and social care needs, the total organisation of work associated with meeting those needs, plus the impact on those involved with that work and its organisation’ [10]. They provide a framework for the understanding of the linkages between individual trajectories of care and broader health and social care systems.

Many elderly patients with multiple health problems perceive health services as complex and challenging to comprehend, and therefore need support from health care professionals to ensure continuity of services. The transition from hospital to home can be an uncertain and challenging experience [11–13]. Information and participation in planning and decision-making during hospital stays and discharge may be inadequate; therefore, elderly patients should be encouraged to participate. Studies have shown that patients’ health needs must be considered and the hospital environment should be organised and prepared to encourage patients’ participation in their discharge planning [14–17].

International as well as Norway health authorities have deployed standardised care pathways for specific patient groups. However, in Norway, no care pathways have been fully established and understood for elderly patients with multiple health problems [18]. Local tailoring combined with standardisation can be important in developing pathways that enable different purposes and contexts [19–21].

Research has emphasised the need to expand our understanding of complex care trajectories and why integrated health and social service care can be challenging. The importance of investigating how individual activities and decisions take place in an organisational context and how involved persons interact has also been emphasised [22].

The perspectives of elderly patients with complex health problems of care trajectories have been insufficiently described in previous studies [8, 19, 23, 24]. New insights are required to achieve an integrated care pathway. Therefore, this study aims to explore how elderly patients with complex health problems engage in and interact with their care trajectories across different health care systems where several health personnel are involved.

## Methods

The study used an explorative design. We adopted a qualitative multi-case approach to obtain an in-depth understanding of patients’ perspectives of care trajectories and how patients participate during their hospital stay, discharge and return to home process. This case approach was considered appropriate for examining patients’ real care trajectories because it was possible to account for the diversity of context [25]. The multi-case method enabled the exploration of inequalities and similarities across care trajectories, aiming to identify common patterns [26]. We recruited 11 patients representing diversity across contexts for our data collection. For each case, observations and multiple interviews were carried out to elucidate the divergent aspects of care trajectories. The cases provided us with rich and comprehensive information relevant to the aim of this study [25].

### Setting and participants

The Norwegian health care system consists of two organisational structures. The local municipalities are responsible for providing primary care services, including general practitioners (GPs), intercommunal emerging primary care centres, home care services, nursing homes and preventive services. The Ministry of Health and Care Services is responsible for specialist care, which involves all hospitals. In 2012, the government implemented the Norwegian Coordination Reform [27] to strengthen the interaction between different levels of health services and to secure coordinated health care. Development of integrated care pathways, especially for patients with long-lasting complex health needs, has increased the focus on developing pathways. This reform, combined with the Patients Right Act, emphasises the importance of patient participation in improving the continuity and quality of care.

To identify patients who met our inclusion criteria, the study’s starting point was conducted at two different hospitals located in the same health region: one rural and one urban hospital. We intended to follow patients during their hospital stays and trajectories across different health care levels. We considered it inappropriate to recruit patient participants prior to possible hospital admissions. The recruiting process was, therefore, conducted at the hospital departments.

Patients were selected from the surgery and internal medicine departments of the hospitals. The inclusion criteria for the patient participants were as follows: older than 65 years, having 2 or more chronic diseases and living at home before hospital admission. The exclusion criteria were if the patient was not capable of giving consent or in the terminal phase. A contact nurse in the eligible departments informed the patients verbally and in writing about the study. Eighteen patients were requested for participation. Eleven patients consented to participate whereas seven patients declined due to worsening health conditions. The patients varied in age and the distance between their homes and the hospitals. Patients from nine different municipalities were involved. The population ranged from 2000 to 27.000 inhabitants. Characteristics of the patients who agreed to participate and the observation period for each patient are shown in Table 1. No participants dropped out of the study.

**Table 1:** Participants, observation periods, number of interviews and distances between home and hospital

Participant ^a^)	Age ranges^b^)	Observation period	Number of interviews	Distance between home and hospital
Finn	1	7 weeks	2	175 km
Maria	1	2 weeks	2	5 km
Eva	2	7 weeks	3	30 km
Eric	2	18 weeks	4	40 km
Anna	2	6 weeks	3	5 km
Kai	2	5 weeks	2	80 km
May	3	13 weeks	4	5 km
Martha	3	7 weeks	1	200 km
Albert	3	2 weeks	1	10 km
Henry	3	3 weeks	1	200 km
Hannah	3	10 weeks	1	20 km

^a^)The participants names are pseudonyms

^b^) The participants are presented in three age range groups. Group 1, range between 65 and 70, group 2, range between 71 and 80 and group 3, range between 81 and 95

### Data collection

We applied an observationally driven approach to this case study [28]. The starting point for the data collection was to meet the patient in the department where he or she was hospitalized. The first author (MK), a PhD candidate and an experienced geriatric nurse, conducted field notes and conversations with the patients and repeated more structured interviews with the patients during the observation period. The professional background of the researcher was known to the participants. Moderate participant observation was used; the researcher was identifiable, interacted with the participants and engaged in activities, but did not participate in the setting [29].

The focus of the observations was to identify situations and activities during the care trajectory in connection with the health services and patient’s interactions with the involved persons. Typically, observation points at the hospitals involved sitting with the patient and observing activities and dialogue between the patient and health personnel, observing morning meetings with the staff group, noting pre-visits and doctors’ attendance at the patients’ rooms and following patients during discharge and their transfer home. In the municipalities, the observations commenced at the professional base of the homecare nursing or the multidisciplinary team and following the staff on their visits to the patients’ homes. On some days, when the first author visited patients at their homes or rehabilitation units, the next of kin was also present. The length of the structured interview varied from 5 to 45 min, according to the patient’s health status and day-to-day condition. The main theme of the interview was on the patient’s past, current, and future perspective on the care trajectory (See additional file 1). Overall, the first author conducted 24 structured interviews and 86 h of observations. The data were collected from November 2017 to June 2018.

### Analysis process

The first author transcribed all the recorded interviews verbatim. Field notes were written down as short sentences during the observation. Immediately after the observations, the field notes were expanded into full sentences. All the data was de-personalised before analysis. A thematic analysis approach using Braun and Clarke’s [30] was applied*.* Initially, the first author read the field notes and the interviews thoroughly and chronologically for each case to identify essential characteristics and patterns. Notes were taken to describe the descriptive and analytical attributes of the data. Thereafter, the data were read and coded systematically and the codes were organised into possible sub-themes for the entire cases as illustrated in Table 2. The back-and-forth process between the codes and possible themes involved reviewing relevant research and theoretical perspectives to help understand the data. This process revealed three main themes.

**Table 2:** Example of the analysis process

Example of codes	Example from codes to sub-themes	Identified themes
It’s better to re-housing than move to a nursing home	To looking for alternatives and be flexible	Continuous consideration of opportunities and alternatives
Consider to stay at home or move to relatives	To have long and - short perspectives	
To get in better shape and see what happens	To use their strength appropriately	
	To sought for the most important health personnel	Consideration for appropriate alliances
The importance of previous contact with and trust in health professionals	To describe my social network	
Despair by not knowing who to contact	To use my long relation with the health personnel	
What I can’t do anything about, is not worth the effort	To deal with unresolved question and unclear responsibilities	Circumvention of the health care initiation of planned steps
Looking for the best way to react	To consider the access to health care	
Many unresolved questions and considerations at the same time	To choose another approach to handle the further direction of the care trajectory	
Organizing and coordinating further treatment and follow-up		
Searching for practical solutions that contribute to alternatives		
Not well-facilitated in the environment for participating		

### Ethical considerations

The study has been notified by the Norwegian Centre for Research Data (ID: 54551) and assessed and approved by the hospital data controller of the two hospitals.

Participation in this research was based on informed, voluntary consent. Ethical issues related to consent were considered during the recruitment process. During the observation period, the first author had a special awareness of maintaining voluntary and consent-based participation. Information about the possibilities to withdraw any time from the study was given both verbally and in writing. The first author had no contact with the patients before they were informed of this study by the nurse. The patients’ consent to participate was given both verbally and in writing. To ensure that patient anonymity is protected, some of the demographic data were rewritten. Hospitals and municipalities involved in the studies were anonymised.

## Results

The overall findings of this study suggest that patients’ engagement in managing their care during the care trajectory is not a linear process regarding time and space or situations and events that need action. They chose a variety of strategies to participate in their care management, driving the care trajectory forward and handling barriers. The patients were engaged and positioned themselves according to three identified themes: continuous consideration of opportunities and alternatives, consideration for appropriate alliances and circumvention of the health care initiation of planned steps.

The analyses revealed that the care trajectory is characterised as a landscape of complex and interconnected events and situations—sometimes planned, chaotic or ad hoc. In some settings during the care trajectory, patients need to deal with many activities simultaneously. The observations revealed that, in some situations, patients had to manage information on the follow-up treatment, medication changes, decisions regarding further health care and readiness to return home or nursing homes, which was given at the same time.

Another simultaneous event that occurred during hospital stays was when health personnel decided to move a patient to another ward or unit at the hospital because of limited space while they were prepared for discharge. Such a situation could be sudden and unexpected to the patient. On the day of discharge from the hospital, several activities, such as ongoing treatment and various controls, were conducted. In the municipality, the patient could receive health care services from several units with different health personnel involved, including homecare nursing and home care assistance, multidisciplinary team, physiotherapists and GPs. Parallel to primary care health services, patients also received outpatient treatment at the hospital.

To provide an in-depth understanding of the themes, cases that are typically for each theme are chosen.

### Continuous consideration of options and alternatives

A strategy some patients used was to continuously consider options and possibilities on how they managed different actions and challenges during their care trajectory. The patients expressed their views on their current health situations. Furthermore, they also questioned how they could manage their situations and use their strength and energy appropriately and weighed different possibilities. They consider what was most important, what could wait and what was not possible.

Sometimes patients felt they were not ready to make decisions concerning changes in their housing situation or plan for further health care. They chose to see ‘what happens’ and prolong the decision. In situations involving several individuals and rapid changes in care environments, patients deliberated about their strength and capacity and assumed a distant or observant position.

Below, we chose to present two typical cases that describe the patients’ considerations of their opportunities and alternatives to housing conditions and further health care. The patients needed long-term decision-making beyond the period of hospitalisation and discharge planning. They held off on deciding until they were ready for it.

#### Case

Anna was admitted to an internal unit at the local hospital due to chronic breathing difficulty that worsened. Anna lived in her apartment in a community near the hospital. A homecare nurse visited her once a day; and during the rest of the day, Anna managed by on her own. In the early phase of her stay at the hospital, she expressed that she was afraid she could no longer manage by herself at home; her health condition was too poor. However, she was still looking for possible options for going home and thinking about what she might need in terms of health care and facilitation, such as night visits by homecare nurses. This option was important for her, as it made her feel safe about being alone at home.

A few days after hospitalization, Anna was discharged to a rehabilitation unit in her home community. During her stay at the hospital and the rehabilitation unit, there was a conversation between her and the health personnel about either being discharged to her apartment or being moved to a nursing home. Anna was reluctant to be active in these decisions*.* Several times during these weeks, she expressed that she had to be in better shape and wait for further development before making a decision as illustrated in this quote:

‘When I feel that I can’t manage myself at home, there is no point in trying. Then, I just have to get help from someone by applying for a permanent place in a nursing home or a sheltered house. However, I have to say I am not ready for that yet. If I do not get any better, then I will have no choice, but I have to decide on that later. I will take it one day at a time and see what happens*.’*

After 3 weeks at the rehabilitation unit, Anna expressed that she needed to take it 1 day at a time but could already take a more active position:

‘I still have problems with my breath, but I am so satisfied and feel I am in better shape. I know my body. Next week, I will go home with help from homecare nurses. Tomorrow, we are going to have a meeting here. Then, we will decide on the number of visits I will need from the homecare nurse. Then, I will know. We are going to have the meeting together with the leader at the unit.*’*

Anna’s case shows how several patients constantly considered their capacity and strength and continuously searched for possibilities and options. Anna chose to wait and hold off on deciding whether she should return home or to a nursing home.

#### Case

May considered changing the house conditions to achieve the appropriate level of care for herself and her husband. She lived with her husband, who received assistance every day from homecare nurses due to illness and functional decline. They lived in a single house with bedrooms on the second floor. May took care of housekeeping, organised health care, and kept in touch with their GP and homecare nurses, among others. I (first author) met May when she was admitted to a hospital because of vertigo and declining general conditions, and followed her during her hospital stay and some months after her return home. After her discharge, she and her husband started receiving additional homecare nursing assistance, and a personal emergency response system was installed in their home. She worried that she and her husband could fall down their stairs. She mentioned several times that she and her husband were discussing applying for sheltered housing. According to May, the health personnel in community care told them many times that they could move to a sheltered house. She expressed:

‘We intend to apply for it, but we have not chosen to do so yet. Now, life goes on as before. It's stable, and I’ve got a personal emergency response system. The neighbour picks up the mail for us. Basically, we do not want to move out of the house as long as we can manage to lock the door!’

The cases show the constant considerations of what options would be the best for them.

### Consideration of appropriate alliance partners

One strategy that patients used to handle unclear situations and considerations of health care was to search for health personnel they found trustworthy who could help them organise their health care needs. The patients described the people that supported them in their daily living and the trustworthiness of the health personal. These trusted persons and health personnel were strong alliances for patients during their care trajectories.

The following case describes how a patient actively searched for health care personnel who could help or take responsibility in his situation, which involved persistent health problems.

#### Case

Eric was a patient with a complicated and persistent illness. After spending several weeks in a hospital for diagnosis and treatment, he was discharged and sent home. He lived with his wife in an apartment. Eric followed-up with two different wards at the hospital and received homecare nursing and physical therapy from the municipal health service. In daily life, he expressed that he and his wife had many unanswered questions about his health problems and symptoms. Eric mentioned several times how challenging it was to find health care personnel at the hospital who could give accurate information and somebody who could be responsible for his ongoing medical treatment. He was told that he needed to contact his GP, but he felt his GP was not particularly involved. Due to his limited interaction with his GP, Eric felt his symptoms were initially not taken seriously, and he lost trust in his GP. At one point, Eric felt he needed advice related to specific symptoms involving his leg and ongoing treatment but felt that he was not likely to receive proper health care. Thus, he approached a homecare nurse he trusted to contact the doctor at the hospital on his behalf about his concern with the leg. He told he did it this way:

‘The call becomes a priority when the nurse calls to ask about the symptoms. I talked to the nurse about this physiotherapist too, he needs a case summary and referral from the doctor. Now it’s okay, I got this by the doctor when I was at the hospital for treatment this week.’

This case illustrates how a patient actively searched for alliance partners to obtain access to proper health care in a setting where he needed to interact with many actors at different levels.

### Circumventing the health care initiation of planned steps

We also identified cases where patients circumvented the hospitals’ formal planning systems because the situations were not well-facilitated or appropriate for their ability to participate. In some cases, patients design their care trajectory. The following case is an example of how a patient circumvented the hospital’s planning process.

#### Case

Henry was admitted to a hospital because of heart failure. Some complications in his health situation unexpectedly prolonged his hospital stay. Because Henry suffered from hearing loss and slowed speech, it was challenging for him to understand and follow the information that was given to him at the hospital. During the pre-visit, the nurse and doctor discussed Henry’s return home. The nurse announced that Henry would need to establish some home care services, if nothing else, to help with his medication. During his doctor’s patient rounds, Henry did not have sufficient time to ask questions or give feedback. Henry tries to tell the doctor he has some questions, but it takes time because of his trouble with the speech. After a few seconds, the doctor says he can contact a nurse when he remembers.

After the visit, Henry told me (first author) that he was unsure about what the doctor meant when he told Henry that he should stay for at least one more day, that is, whether it meant that he might return home the next day or not.

Henry lived with his wife in a single house located in a rural area far from the hospital. His next of kin and health personnel from the community could not visit him during his hospital stay. He described his lasting relationship with the leader of the local homecare nursing facility and his GP. He expressed trust in the local health service like this.

‘I regularly visit my GP to take blood samples. I think my doctor is very capable. The leader in the homecare facility is a decent person. He knows about everything. He has helped us several times.’

During his hospital stay, Henry spoke of having phone contact with the leader of the homecare nursing facility. Together, they organised his need for health care and the assistance that he would require after discharge. He also contacted a neighbour to take care of snow shovelling at his home. The leader of the homecare nursing facility stated that he had known Henry and his wife for a long time. He added that Henry had been clear about coming home instead of being transferred to a nursing home. According to the leader, phone contact served as a way to stay in contact with the patient during the latter’s hospital stay.

This case is an example of how a patient actively chose another approach to handle the further direction of his care trajectory. The hospital’s environment and discharge planning did not functionally allow Henry to interact with health personnel. The next of kin could not be near the hospital for support. Therefore, he sought a new option for handling his situation and circumvented the hospital personnel’s plans and processes for discharge.

## Discussion

How the patients were engaged with and interacted in their care trajectories varied and was influenced by their health conditions and how their situation afterward could be managed. We found that the patients, who are often described as vulnerable, carried out considerable ‘homework’ to navigate their health condition as well as the system they accounted [31]. The patients constantly considered opportunities and alternatives in interaction, negotiations and relationships between many actors, or ‘players’ [22] for handling the different challenges and situations that occurred during their care trajectory. To understand why and how they searched for appropriate alliance partners to support them, and in some situations, how they circumvented the planned steps and took different directions in the care trajectory will be discussed against the conceptualisation of care trajectory game (CTG) [22]. The CTG framework merges Strauss et al.’s [32] descriptions on illness trajectories and Elias’s [33] game model and provide a framework to understand and address the dynamics and complexity in the system and thus, move away of thinking trajectories in mono-causal explanations which appear to be the characteristics of current policy [3, 8].

The complexity in the patients’ care trajectory became visible throughout the patient’s multiple considerations about options and multiple alternatives they needed to take into account. They were dealing with balancing their strength and capacity and the complexity of the health care system in how they could be involved in decision-making. Their considerations seemed to be a continuous process. We identified that patient participation in their care trajectory was not linked to specific times or situations. Issues regarding the need for necessary health care and life modifications or changes in living arrangements were deliberated for patients throughout their entire hospital stays and continued after discharge. It was often an ongoing negotiation between patients, health personnel and next of kin. Patients wanted to have options, but time for recovery was often essential in preparing them for participation in decision-making. The patients’ also seemed to keep a watchful waiting whereby they try to maintain the status quo to desired preferences for as long as possible. The patients in this study used different strategies in situations with a disagreement between their preferences, health care need and initiation of planned steps. For example, they waited to be ready for decisions, circumvented planned steps and found a new direction in their trajectory. Allen et al. [22] point on that ‘disagreement’ over plan for further direction in the care trajectory not necessarily needs to be negative for the patient. The negotiations and different input from the involved can bring new opportunities and options, which are more in line with the patients’ preferences. It is not appropriate to try to simplify the complex care trajectories, but rather organize the services so that several opportunities and alternatives can be included [22]. Today’s health care system is characterised by overall expectations to the health personnel to working quickly and efficiently in bed administration, and hospital period is shortening [34, 35]. With reference to CTG, health professionals can form an alliance to achieve an effective transfer of care. As an example to press for a nursing home placement rather than a home discharge, that can be easier to organize. From a health personnel perspective can this solution simplify the complexity in the organization of the patient care trajectories, but on the other side lock and hinder the patient’s ability to see different opportunities and alternatives, which are in preference to the patient’s wishes [22].

Our findings describe situations with interactions between patients and many health personnel at different health services levels. These situations increased the patients’ perceived considerations regarding which personnel could take responsibility for their treatment and organisation of their care. Existing literature has described patients’ and next of kin’ experiences of fragmentation regarding obtaining control and access to the health care system during discharge and follow-up care, which are in line with our findings [36–38]. We found that to handle fragmentation and uncertainty about health care, the patients sought alliance partners who could help in their interaction with and access to health care. When complexity increases in the care trajectory, fragmentation increases between the involved actors, leading to a re-grouping of those involved [22]. Unanswered questions about health problems and symptoms were uncertainty patients in our study experience and a lack of available and appropriate information. Kneck et al. [39] have pointed out that the patient is expected to be an active partner, but that the patients at home can have insufficient information to manage their illness. They may be unsure of ‘which symptoms might occur and who to contact for different needs’ [39]. Mattingly et al. [31] use the term ‘chronic homework, about tasks the patients and family caregivers are expected to carry out when moving health care from hospital to home. Their supporting network was essential to handle this ‘homework’, and to strengthen the patient’s possibility to take responsibility for their care. In our study, the patients described how they used their alliance partner strategically as an important support to achieve access to health care and to drive the plan further in the trajectory.

When the patients experienced that they were not involved in decisions concerning themselves, they used their strategies to circumvent the system. Insufficient facilitation of patient participation in the care environment is another barrier described in our study. Time and space for patients to participate in discussions about their health and the need for health care were not always arranged properly. Despite these situations, the patients considered their possibilities and alternatives and circumvented barriers and make their further plans*.* According to CTG, the resources available can both shape the complexity and cause those involved to make various moves to circumvent barriers.

Findings in our study describe inadequate facilitation of participation and necessary access to health care, norms and the view of the elderly person may contribute to it. Health professionals’ views of the elderly and younger are highlighted as a possible challenge in access to treatment and follow-up [40, 41]. Hamran et al. [40] found that only based on a norm understanding that ‘they are just old’ access to health care could be less, and the time for treatment and improvement was expected to be resolved in the same way as young people who do not have the same complexity. Norms and values are interviewing with the actions and decisions in the care trajectory and increases complexity [22].

### Implication for practice

For an elderly patient with complex health problems, there is expedient to develop a care trajectory that is developed to meet the need for flexibility. In practice, it can mean accepting that the patient is about participating in managing and making decisions, often a continuous and long-term process. Facilitating for this in organizing the health system service, and time and space for the patients’ considerations to managing necessary modifications in everyday life such as re-housing or move to a nursing home.

### Methodological strengths and limitations

Triangulation of data sources, observations and individual interviews were used to investigate the care trajectory from different perspectives and settings, which was appropriate given then intention of the current study to gain a richer and deeper insight of patients’ care trajectories.

The first author who conducted the observations and interviews was an experienced geriatric nurse. The researcher’s assumption, skills and knowledge will influence the focus in observations and shape the interpretation [42]. Background as a nurse gave the advantage to understand the field. To strengthen the trustworthiness, additional reflection notes were performed describing choices, questions or thoughts that arose during the observations and were for review by the research group.

The first author followed each patient over a long period, which contributed to a broad understanding of each patient’s case. We experienced some challenges in recruiting participants due to patients’ health conditions and vulnerable situations. Despite that the participants’ age, living situation and home setting varied. Since the findings are based on a small sample, they should be considered with caution in the light of generalizability. Nevertheless, we believe these findings provide new insight and understanding of the complexity of elderly patients’ care trajectories.

## Conclusion

The patients’ considerations of their health care needs and adjustments to living arrangements are constant throughout the care trajectory. These considerations are often long term, and the patients engagement in and management of their care trajectory is not associated with particular times or situations. It may be important for elderly patients’ time for recovery in order to consider different possibilities and options before managing necessary modifications in everyday life.

Disagreements between preferences, the need for health care and the initiation of planned steps, leads to different strategies from the patients. They wait to be ready for decisions, circumvent planned steps and find a new direction in their trajectory.

Achieving consistency between the health care system and the patient’s pace in the decision-making process during the care trajectory, may lead to a more appropriate level of health care in line with the patient’s preferences.

## Supplementary information

**Additional file 1.** Interview guide. Patient interview schedule

## Abbreviations

GP: General practitioner
CTG: Care trajectory game
