# Supplementary material for: Elderly patients with complex health problems in the care trajectory: a qualitative case study
Source: BMC Health Serv Res. 2020 Jun 29;20:595. doi: 10.1186/s12913-020-05437-6 (PMC7325247; doi:10.1186/s12913-020-05437-6)
Supplement: Supplementary file 1 — Additional file 1. Interview guide. Patient interview schedule [file 12913_2020_5437_MOESM1_ESM.docx]

Patient interview guide

The main theme of the interview was the patient’s past, current, and future perspective on the care trajectory.

| Topics related to the hospitalization |
| --- |
| Can you please tell me about the days before you were admitted to the hospital and what happened then?   - What changes did you experience with respect to your need for health care? - Please tell me about the health care you received? - Please tell me how you experienced the health care you had received? - Please tell me what you think will happen after you discharge from the hospital? |
| Topics related to the time period after the hospitalization. |
| Can you please tell me about the time you spent at hospitals and the period after your discharge from the hospital?   - How do you experience your situation now compared to before the hospitalization? - Please tell me about the health care you are receiving now? - Please tell me about what you think will happen next? |
